# Association of *ACE2* and *TMPRSS2* genes variants with disease severity and most important biomarkers in COVID-19 patients in Bosnia and Herzegovina

**DOI:** 10.3325/cmj.2024.65.220

**Published:** 2024-06

**Authors:** Neven Meseldžić, Besim Prnjavorac, Tanja Dujić, Maja Malenica, Una Glamočlija, Lejla Prnjavorac, Omer Bedak, Selma Imamović Kadrić, Damir Marjanović, Tamer Bego

**Affiliations:** 1 Department of Pharmaceutical Biochemistry and Laboratory Diagnostics, Faculty of Pharmacy, University of Sarajevo, Sarajevo, Bosnia and Herzegovina; 2 General Hospital Tešanj, Tešanj, Bosnia and Herzegovina; 3 Department of Genetics and Bioengineering, International Burch University, Sarajevo, Bosnia and Herzegovina; 4 Institute for Anthropological Research, Zagreb, Croatia; 5 Faculty of Biotechnology and Drug Development, University of Rijeka, Rijeka, Croatia

## Abstract

**Aim:**

To assess the association of single nucleotide polymorphisms (SNPs) in the *ACE2* and *TMPRSS2* genes with COVID-19 severity and key biomarkers.

**Methods:**

The study involved 750 COVID-19 patients from Bosnia and Herzegovina, divided into three groups: mild, moderate, and severe cases. Genetic variations within the *ACE2* (rs2285666) and *TMPRSS2* (rs2070788) genes were examined with real-time polymerase chain reaction. Biochemical markers were determined with standard procedures.

**Results:**

There was a significant difference in the rs2070788 genotype distribution between patients with mild and moderate symptoms, but not between other groups. For the rs2285666 polymorphism, no significant difference in genotype distribution was found. In patients with mild symptoms, carriers of the GG genotype of rs2070788 had significantly higher total bilirubin levels than carriers of the AA genotype. Similarly, carriers of the TT genotype of rs2285666 had significantly higher activated partial thromboplastin time and international normalized ratio, and lower lactate dehydrogenase levels compared with the CC genotype. Among patients with severe symptoms, carriers of the GG genotype showed significantly higher potassium levels than carriers of the AA genotype, while carriers of the TT genotype showed significantly higher erythrocyte count as well as hemoglobin and hematocrit levels compared with the CC genotype.

**Conclusion:**

This study highlights the role of genetic factors, particularly SNPs in the *ACE2* and *TMPRSS2* genes, in determining COVID-19 severity, aiding patient risk assessment and prognosis.

COVID-19 is a viral illness caused by the severe acute respiratory syndrome coronavirus 2 (SARS-CoV-2) (1). Its presentation ranges from asymptomatic cases to severe manifestations, such as respiratory failure and death. Since its emergence in December 2019, COVID-19 has claimed over 6.5 million lives globally (2). Several risk factors have been identified for severe COVID-19, including male sex, older age, specific ethnicities, obesity, and cardiovascular and respiratory disorders. Additionally, host genetic factors have demonstrated a significant influence on susceptibility to infection and disease severity (3). The invasion of host cells by SARS-CoV-2 depends on the presence of two key factors: angiotensin-converting enzyme 2 (ACE2) and transmembrane protease serine 2 (TMPRSS2). ACE2 serves as the receptor through which the virus enters the host cells, while TMPRSS2 facilitates the priming and activation of the viral spike protein, enabling viral fusion and entry (4). Coronaviruses employ the spike (S) protein to enable their entry into specific cells. In the case of SARS-CoV-2, the S protein engages ACE2 as the entry receptor, while the cellular serine protease TMPRSS2 is responsible for priming the S protein (5). Due to the significance of ACE2 and TMPRSS2 in this process, it is important to understand the genetic variations in *ACE2* and *TMPRSS2* and their association with COVID-19 outcomes.

ACE2 is a carboxypeptidase consisting of 805 amino acids, responsible for the removal of a single amino acid from the C-terminus of specific substrates. It plays a crucial role in the renin-angiotensin-aldosterone system, which is integral to normal physiological functions (6,7). In this system, ACE2 transforms angiotensin I and angiotensin II, which are produced by renin and ACE, respectively, into angiotensin 1-9 and angiotensin 1-7. A multitude of conflicting studies explored the effects and prevalence of genetic variations within the *ACE2* gene and their association with COVID-19. This discrepancy has motivated us to embark on a similar study focusing on the population of Bosnia and Herzegovina (8-12).

TMPRSS2 is a transmembrane protein of the type-II category, known for its serine protease activity. While the precise physiological role and substrate specificity of TMPRSS2 are not yet fully understood, its significance in the context of respiratory virus infections, particularly influenza viruses and SARS coronaviruses, is well-established. TMPRSS2 is predominantly found in the epithelial cells of the gastrointestinal, respiratory, and urogenital systems (13). Notably, three primary cell types share the co-expression of TMPRSS2 and ACE2: type II pneumocytes, ileal absorptive enterocytes, and nasal goblet secretory cells. Although ACE2 is typically found in the lower airway, its expression is higher in the upper airway. As a result, numerous airway cells expressing ACE2 also concurrently express TMPRSS2, which suggests their involvement in viral entry and infection processes (14). Based on all this, we can assume that ACE2 and TMPRSS2 are key factors in COVID-19 infection because they facilitate the virus's ability to enter and infect human cells. Understanding the role of these proteins is crucial for developing treatments and interventions to mitigate the spread of the virus and its impact on the human body.

Single nucleotide polymorphisms (SNPs) significantly affect the interaction between microbial agents and host cells, the body's resistance to infection, and the manifestation of severe disease symptoms. Genetic variations in the *ACE2* and *TMPRSS2* genotypes have been observed to influence the outcome and progression of COVID-19. Numerous studies have revealed that the frequencies of certain alleles and SNPs in these genes are associated with differences in COVID-19 prevalence among various ethnic groups (8-12). Hence, our primary aim was to investigate the potential association between ACE2 (rs2285666 C>T) and TMPRSS2 (rs2070788 A>G) and disease severity and crucial biomarkers in COVID-19 patients.

## Materials and methods

### Study population

The study enrolled patients who tested positive for SARS-CoV-2 (confirmed by real-time polymerase chain reaction [RT-PCR] test) upon their admission to the General Hospital Tešanj, Bosnia and Herzegovina, between January and July 2021. The patients (n = 750) were classified into three groups based on their clinical signs, symptoms, radiological and laboratory findings, and overall condition, following established guidelines for the classification of COVID-19 patients (15): mild, moderate, and severe. The assessment was conducted by clinicians who were guided by widely accepted protocols for patient classification. The research adhered to the ethical principles outlined in the Declaration of Helsinki for Medical Research Involving Human Subjects. The study protocol was approved by the Ethics Committee of the General Hospital Tešanj (01-4-18/21, 11th January 2021) and Ethics Committee of the Faculty of Pharmacy, University of Sarajevo (0101-3221/22, 13th June 2022). All participants provided written informed consent before participating in the study.

### Samples collection

Blood specimens were collected by standard venipuncture. After coagulation at room temperature for 30 min, serum was separated by centrifugation at 3000 g for 15 min and stored at -20 °C until analysis. Serum samples were aliquoted and stored to ensure their availability for specialized analyses conducted as part of this project.

### Biochemical and hematological measurements

All biochemical parameters were analyzed using International Federation of Clinical Chemistry and Laboratory Medicine standard protocols. The serum levels of the biochemical parameters were determined by using Biotecnica BT3500 autoanalyzer (Biotecnica Instruments S.p.A., Rome, Italy). Hematological measurements in whole blood were conducted by using Mindray BC 3200 (Shenzhen Mindray Bio-Medical Electronics Co., Ltd, Shenzhen, China); D-dimer levels were measured by using Biomerieux VIDAS (BioMérieux Clinical Diagnostics, Marcy-l'Étoile, France); prothrombin time and activated partial thromboplastin time (aPTT) by using Stago – STart (Diagnostica Stago, S.A.S., Asnières sur Seine, France); while sodium and potassium levels were measured by using Seac-Radim Fp20 (Radim Diagnostics, Pomezia, Italy). The acid-base status was determined with ABL800 FLEX (Radiometer, Copenhagen, Denmark) blood gas analyzer.

### Genetic testing

Blood samples were collected from the antecubital vein into siliconized tubes with EDTA (BD Vacutainer Systems, Plymouth, UK) and stored at -20 °C. For the isolation of genomic DNA, E.Z.N.A.® DNA isolation kit (Omega Bio-tek, Inc., GA, USA) was used. The purity and concentration of isolated DNA were determined by UV/VIS spectrophotometer BioSpec-nano (Shimadzu Corp. Kyoto, Japan). After extraction, DNA samples were stored at -20 °C. *ACE2* (rs2285666 C>T; ID C___2551626_1_) and *TMPRSS2* (rs2070788 A>G; ID C___2592038_1_) gene polymorphisms were genotyped with hydrolysis probes and RT-PCR using TaqMan® SNP Genotyping Assays (Applied Biosystems, Foster City, CA, USA). The QuantStudio® 5 RT-PCR System was used for the genotyping process (Applied Biosystems).

### Statistical analysis

The normality of data distribution was tested with a Kolmogorov-Smirnov test and Shapiro-Wilk test. The variables that were not normally distributed were log-transformed. χ^2^ test was applied to examine the differences in allele frequencies and genotype distributions among the three groups of patients. Logistic regression analysis was used to assess the association of gene polymorphism with the severity of COVID-19, after adjustments for age and sex. Odds ratios (OR) with confidence intervals (95% CI) were calculated. The significance of differences in biochemical and hematological measurements according to genotypes of analyzed polymorphisms was estimated with ANOVA (Bonferroni test). A *P* value ≤0.05 was considered statistically significant. Statistical analysis was performed with SPSS, version 26.0 (IBM, Armonk, NY, USA).

## Results

Patients with mild clinical symptoms had a mean age of 58.0 years (SD ±16.1), with 56.5% of them being male. Patients with moderate disease severity had a mean age of 64.4 years (SD ±12.3), with 54.4% being male, while patients with severe clinical symptoms had a mean age of 67.8 years (SD ±13.1), with 57.2% being male (Table 1). The *TMPRSS2* gene polymorphism rs2070788 showed a significant difference in genotype frequencies between patients with mild and moderate disease (Table 2).

**Table 1:** Patients’ demographic data

	Group		
	mild	moderate	severe
Total patients (n = 750)	262	252	236
Age (mean ± SD)	58.0 ± 16.1	64.4 ± 12.3	67.8 ± 13.1
Men (n = 420)	148	137	135
Age (men) (mean ± SD)	58.2 ± 16.5	63.1 ± 12.1	65.9 ± 13.3
Women (n = 330)	114	115	101
Age (women) (mean ± SD)	57.7 ± 15.7	65.7 ± 12.5	70.2 ± 12.5

**Table 2:** Genotype and variant allele frequencies for the *ACE2* gene polymorphism rs2285666: C>T and *TMPRSS2* gene polymorphism rs2070788: A>G

Polymorphism	Genotype	Mild	Mutated allele frequency	Moderate	Mutated allele frequency	Severe	Mutated allele frequency	P*
rs2285666	C/C	193 (73.7%)	0.20	182 (72.2%)	0.21	171 (72.8%)	0.19	Mild/Moderate = 0.696
	C/T	31 (11.8%)		36 (14.3%)		40 (17.0%)		Mild/Severe = 0.124
	T/T	38 (14.5%)		34 (13.5%)		24 (10.2%)		Moderate/Severe = 0.430
	**Total**	262	P1^†^<0.001	252	P2^‡^<0.001	235	P3^§^<0.001	0.369
rs2070788	A/A	83 (31.7%)	0.46	53 (21.0%)	0.52	68 (28.9%)	0.49	Mild/Moderate = 0.020
	A/G	116 (44.3%)		135 (53.6%)		102 (43.4%)		Mild/Severe = 0.620
	G/G	63 (24.0%)		64 (25.4%)		65 (27.7%)		Moderate/Severe = 0.053
	**Total**	262	P1 = 0.209	252	P2 = 0.507	235	P3 = 0.130	**0.046**

*the significance level of the χ^2^ test for the comparison of genotype frequencies between mild, moderate and severe group of COVID-19 patients.

†P1 – value for Hardy-Weinberg equilibrium for COVID-19 patients with mild disease severity.

‡P2 – value for Hardy-Weinberg equilibrium for COVID-19 patients with moderate disease severity.

§P3 – value for Hardy-Weinberg equilibrium for COVID-19 patients with severe clinical outcome.

### *ACE2 gene polymorphism* (rs2285666)

The allele frequencies of *ACE2* gene polymorphism rs2285666 C>T were not in Hardy-Weinberg equilibrium (HWE) (*P* < 0.05). No significant differences in the genotype frequencies of the *ACE2* gene polymorphism were found between these 3 groups of patients (*P* > 0.05). Logistic regression adjusted for age and sex did not confirm the expected association of *ACE2* gene polymorphism with the severity of COVID-19 (OR = 0.73, 95% CI 0.44–1.21, *P* = 0.221) (Table 3). Among COVID-19 patients with mild disease severity (Table 4), individuals carrying the risk TT genotype of rs2285666 displayed significantly higher aPTT (*P* = 0.008), international normalized ratio (INR) (*P* = 0.036), and lower lactate dehydrogenase (LDH) levels (*P* = 0.038) than those with the CC genotype. Carriers of the TT genotype showed significantly higher creatinine levels (*P* = 0.011) than individuals with the CT genotype. Patients with the CC genotype had significantly higher levels of hemoglobin (*P* = 0.015), hematocrit (*P* = 0.012), creatinine (*P* = 0.001), and creatine kinase (CK) (*P* = 0.043) than carriers of the CT genotype. In patients with moderate disease severity (Table 5), patients with the high-risk TT genotype of rs2285666 showed significantly higher methemoglobin (FMetHb) levels (*P* = 0.013) than those with the CC genotype. Moreover, carriers of the TT genotype demonstrated significantly higher erythrocyte counts (*P* = 0.002), hemoglobin levels (*P* = 0.001), hematocrit levels (*P* = 0.007), and aPTT levels (*P* = 0.003) than those with the CT genotype. Additionally, individuals with the CC genotype had significantly higher erythrocyte counts (*P* = 0.029), hemoglobin levels (*P* = 0.001), and lower erythrocyte sedimentation rate (ESR) (*P* = 0.006) and FMetHb levels (*P* = 0.048) than carriers of the CT genotype. Among the COVID-19 patients who experienced severe clinical outcomes (Table 6), individuals with the high-risk TT genotype of rs2285666 showed significantly higher erythrocyte counts (*P* = 0.016), hemoglobin levels (*P* = 0.026), and hematocrit levels (*P* = 0.020) than patients with the CC genotype. Moreover, carriers of the TT genotype exhibited significantly higher erythrocyte counts (*P* = 0.005), hemoglobin levels (*P* < 0.001), and hematocrit levels (*P* < 0.0019) than patients with the CT genotype. In contrast, patients with the CC genotype had significantly higher hemoglobin levels (*P* = 0.002), hematocrit levels (*P* = 0.009), and significantly lower glucose levels (*P* = 0.024) than carriers of the CT genotype.

**Table 3:** Association of ACE2 rs2285666 and TMPRSS2 rs2070788 variants with COVID-19 disease severity*

Polymorphism	OR (95% CI)*		
	Additive	Dominant	Recessive
**rs2285666**	1.40 (0.86-2.28)	0.73 (0.44-1.21)	0.98 (0.68-1.41)
	*P* = 0.171	*P* = 0.221	*P* = 0.922
**rs2070788**	0.82 (0.60-1.12)	1.19 (0.84-1.71)	1.11 (0.78-1.57)
	*P* = 0.214	*P* = 0.331	*P* = 0.569

*Logistic regression analysis was used to calculate the odds ratio (OR) with confidence intervals (95% CI) with adjustments for age and sex.

**Table 4:** Comparison of clinical and biochemical characteristics between different genotypes of *ACE2* gene polymorphism rs2285666 in COVID-19 patients with mild disease severity*^†^

	ACE2 rs2285666 C>T					
	CC (n = 193)	CT (n = 31)	TT (n = 38)	*P* value^‡^ CC/CT	*P* value^‡^ CC/TT	*P* value^‡^ CT/TT
Leukocytes (10^9^/L)	6.75 ± 3.32	6.78 ± 3.19	6.62 ± 2.56	1.000	1.000	1.000
Erythrocytes (10^12^/L)	4.46 ± 0.51	4.26 ± 0.49	4.37 ± 0.58	0.141	0.810	1.000
Hemoglobin (g/L)	136.54 ± 19.54	128.03 ± 20.32	133.50 ± 18.85	**0.015**	0.430	0.174
Hematocrit (%)	39.17 ± 4.73	36.64 ± 5.41	38.02 ± 5.37	**0.012**	0.346	0.190
ESR (mm/h)	58.83 ± 38.78	73.17 ± 38.87	65.16 ± 45.11	0.311	1.000	0.730
Platelet count (10^9^/L)	208.16 ± 79.54	227.97 ± 65.29	223.87 ± 105.35	0.293	1.000	1.000
aPTT (sec)	27.91 ± 6.63	28.18 ± 3.44	30.34 ± 6.34	0.323	**0.008**	0.279
PT (sec)	13.00 ± 2.22	12.94 ± 1.45	14.98 ± 5.43	1.000	0.051	0.343
INR	1.09 ± 0.21	1.08 ± 0.17	1.26 ± 0.57	1.000	**0.036**	0.178
CRP (mg/L)	57.93 ± 71.57	45.35 ± 51.83	67.26 ± 75.94	1.000	1.000	1.000
D dimer (mg/L)	1.35 ± 1.59	1.58 ± 1.69	1.09 ± 1.19	0.949	0.621	0.257
Glucose (mmol/L)	8.11 ± 4.28	8.39 ± 4.80	6.84 ± 2.66	1.000	0.210	0.356
Urea (mmol/L)	6.86 ± 2.91	6.40 ± 2.84	7.09 ± 3.29	0.883	1.000	0.610
Creatinine (umol/L)	93.59 ± 41.97	79.32 ± 18.23	91.74 ± 24.90	**0.001**	0.949	**0.011**
Total bilirubin (umol/L)	14.11 ± 6.16	12.74 ± 3.19	17.00 ± 11.18	1.000	0.115	0.065
CK (IU/L)	322.98 ± 820.21	151.87 ± 211.95	172.18 ± 229.79	**0.043**	0.051	0.849
LDH (IU/L)	491.08 ± 225.44	481.48 ± 182.10	407.13 ± 166.35	1.000	**0.038**	0.199
Sodium (mmol/L)	141.02 ± 10.08	141.13 ± 5.18	141.71 ± 3.86	1.000	1.000	1.000
Potassium (mmol/L)	4.29 ± 0.55	4.28 ± 0.55	4.41 ± 0.39	1.000	0.452	0.821
sO_2_ (%)	89.67 ± 7.18	91.31 ± 4.31	90.18 ± 3.39	0.862	1.000	1.000
ctO_2c_ (vol%)	18.87 ± 2.62	18.31 ± 1.85	18.89 ± 3.17	1.000	1.000	1.000
FMetHb (%)	1.26 ± 0.21	1.28 ± 0.18	1.26 ± 0.18	1.000	1.000	1.000

*Abbreviations: ESR – erythrocyte sedimentation rate; aPTT – activated partial thromboplastin time; PT – prothrombin time; INR – international normalized ratio; CRP – C-reactive protein; CK – creatine kinase; LDH – lactate dehydrogenase; sO_2_ – oxygen saturation; ctO2c – sum of oxygen solved in plasma and chemically bound to hemoglobin; FMetHb – fraction of total hemoglobin that is present as methemoglobin (MetHb).

†Values represent mean ± standard deviation.

‡All differences between various genotypes were tested using ANOVA test (Bonferroni post hoc).

**Table 5:** Comparison of clinical and biochemical characteristics between different genotypes of ACE2 gene polymorphism rs2285666 in COVID-19 patients with moderate disease severity*^†^

	ACE2 rs2285666 C>T					
	CC (n = 182)	CT (n = 36)	TT (n = 34)	*P* value^‡^ CC/CT	*P* value^‡^ CC/TT	*P* value^‡^ CT/TT
Leukocytes (10^9^/L)	7.29 ± 3.74	7.09 ± 3.17	7.40 ± 3.62	1.000	1.000	1.000
Erythrocytes (10^12^/L)	4.32 ± 0.49	4.08 ± 0.47	4.50 ± 0.55	**0.029**	0.165	**0.002**
Hemoglobin (g/L)	132.44 ± 17.75	124.11 ± 14.31	136.29 ± 17.44	**0.001**	0.350	**0.001**
Hematocrit (%)	37.91 ± 4.88	35.85 ± 4.06	39.40 ± 5.27	0.060	0.298	**0.007**
ESR (mm/h)	70.73 ± 40.34	90.79 ± 34.79	78.65 ± 39.84	**0.006**	0.369	0.170
Platelet count (10^9^/L)	219.15 ± 91.22	219.58 ± 94.79	200.85 ± 82.83	1.000	0.904	1.000
aPTT (sec)	27.88 ± 6.69	26.03 ± 3.74	29.12 ± 4.75	0.053	0.054	**0.003**
PT (sec)	13.70 ± 3.12	13.06 ± 1.75	14.53 ± 3.26	1.000	0.470	0.198
INR	1.17 ± 0.32	1.08 ± 0.19	1.20 ± 0.33	0.245	1.000	0.218
CRP (mg/L)	99.75 ± 108.09	76.94 ± 67.24	97.09 ± 63.99	1.000	0.775	0.760
D dimer (mg/L)	2.19 ± 2.49	2.69 ± 3.18	2.28 ± 2.68	1.000	1.000	1.000
Glucose (mmol/L)	8.29 ± 4.42	9.31 ± 4.71	8.73 ± 3.91	0.438	1.000	1.000
Urea (mmol/L)	8.66 ± 5.07	8.34 ± 5.33	7.92 ± 3.91	1.000	1.000	1.000
Creatinine (umol/L)	99.30 ± 41.83	92.17 ± 38.31	95.52 ± 29.03	0.562	1.000	1.000
Total bilirubin (umol/L)	16.20 ± 12.62	14.05 ± 8.97	14.55 ± 5.13	0.449	1.000	1.000
CK (IU/L)	232.29 ± 408.03	175.67 ± 192.30	229.82 ± 233.89	1.000	1.000	1.000
LDH (IU/L)	573.09 ± 271.79	601.00 ± 252.06	581.58 ± 250.09	1.000	1.000	1.000
Sodium (mmol/L)	140.36 ± 10.83	141.97 ± 3.91	140.58 ± 3.89	1.000	1.000	1.000
Potassium (mmol/L)	4.31 ± 0.66	4.38 ± 0.70	4.33 ± 0.58	1.000	1.000	1.000
sO_2_ (%)	88.24 ± 7.66	88.19 ± 5.90	88.89 ± 5.15	1.000	1.000	1.000
ctO_2c_ (vol%)	18.28 ± 3.37	17.03 ± 2.37	18.61 ± 2.15	**0.008**	0.594	**0.014**
FMetHb (%)	1.23 ± 0.25	1.32 ± 0.27	1.32 ± 0.19	**0.048**	**0.013**	0.664

*Abbreviations: ESR – erythrocyte sedimentation rate; aPTT – activated partial thromboplastin time; PT – prothrombin time; INR – international normalized ratio; CRP – C-reactive protein; CK – creatine kinase; LDH – lactate dehydrogenase; sO_2_ – oxygen saturation; ctO2c – sum of oxygen solved in plasma and chemically bound to hemoglobin; FMetHb – fraction of total hemoglobin that is present as methemoglobin (MetHb).

†Values represent mean ± standard deviation.

‡All differences between various genotypes were tested using ANOVA test (Bonferroni post hoc).

**Table 6:** Comparison of clinical and biochemical characteristics between different genotypes of ACE2 gene polymorphism rs2285666 in COVID-19 patients with severe clinical outcome*^†^

	ACE2 rs2285666 C>T					
	CC (n = 171)	CT (n = 40)	TT (n = 24)	*P* value^‡^ CC/CT	*P* value^‡^ CC/TT	*P* value^‡^ CT/TT
Leukocytes (10^9^/L)	7.79 ± 4.34	7.00 ± 3.84	7.87 ± 2.88	0.814	1.000	0.668
Erythrocytes (10^12^/L)	4.29 ± 0.59	4.14 ± 0.66	4.61 ± 0.62	0.277	**0.016**	**0.005**
Hemoglobin (g/L)	133.52 ± 19.60	123.70 ± 19.63	142.96 ± 14.69	**0.002**	**0.026**	**<0.001**
Hematocrit (%)	38.09 ± 5.59	35.60 ± 5.35	41.25 ± 5.16	**0.009**	**0.020**	**<0.001**
ESR (mm/h)	73.96 ± 40.38	84.21 ± 43.84	66.61 ± 37.33	0.771	1.000	0.897
Platelet count (10^9^/L)	192.02 ± 81.29	200.20 ± 117.82	191.54 ± 66.45	1.000	1.000	1.000
aPTT (sec)	28.62 ± 5.04	27.43 ± 3.72	28.25 ± 4.37	0.612	1.000	1.000
PT (sec)	16.75 ± 19.91	13.62 ± 3.40	13.19 ± 1.39	1.000	1.000	1.000
INR	1.15 ± 0.29	1.15 ± 0.37	1.12 ± 0.17	1.000	1.000	1.000
CRP (mg/L)	124.81 ± 90.62	107.82 ± 102.99	117.92 ± 90.01	0.107	1.000	0.900
D dimer (mg/L)	2.23 ± 2.29	1.94 ± 2.29	1.97 ± 2.32	1.000	1.000	1.000
Glucose (mmol/L)	9.42 ± 6.19	10.76 ± 5.38	9.16 ± 3.57	**0.024**	0.271	0.250
Urea (mmol/L)	10.67 ± 7.55	10.38 ± 6.12	10.61 ± 6.15	1.000	1.000	1.000
Creatinine (umol/L)	129.44 ± 104.43	121.38 ± 79.67	119.38 ± 51.27	1.000	1.000	1.000
Total bilirubin (umol/L)	16.66 ± 15.08	23.12 ± 49.29	16.35 ± 5.56	1.000	1.000	1.000
CK (IU/L)	354.19 ± 644.77	293.68 ± 575.31	467.75 ± 819.35	1.000	1.000	1.000
LDH (IU/L)	713.40 ± 374.88	892.23 ± 1202.15	716.71 ± 300.78	1.000	1.000	1.000
Sodium (mmol/L)	139.76 ± 4.49	140.10 ± 8.03	138.29 ± 33.86	1.000	0.081	0.172
Potassium (mmol/L)	4.41 ± 0.73	4.21 ± 0.63	4.45 ± 0.92	0.389	1.000	0.716
sO_2_ (%)	84.03 ± 9.44	82.98 ± 9.66	83.41 ± 14.52	1.000	1.000	1.000
ctO_2c_ (vol%)	17.19 ± 3.41	15.55 ± 3.39	17.97 ± 3.28	**0.005**	0.210	**0.003**
FMetHb (%)	1.27 ± 0.24	1.25 ± 0.21	1.24 ± 0.24	1.000	1.000	1.000

*Abbreviations: ESR – erythrocyte sedimentation rate; aPTT – activated partial thromboplastin time; PT – prothrombin time; INR – international normalized ratio; CRP – C-reactive protein; CK – creatine kinase; LDH – lactate dehydrogenase; sO_2_ – oxygen saturation; ctO2c – sum of oxygen solved in plasma and chemically bound to hemoglobin; FMetHb – fraction of total hemoglobin that is present as methemoglobin (MetHb).

†Values represent mean ± standard deviation.

‡All differences between various genotypes were tested using ANOVA test (Bonferroni post hoc).

### TMPRSS2 gene polymorphism (rs2070788)

The allele frequencies of the *TMPRSS2* gene polymorphism rs2070788 A>G were in HWE in all patient groups (*P* > 0.05). Significant differences (*P* = 0.046) in genotype frequencies of the *TMPRSS2* gene polymorphism were found between patients with mild and moderate symptoms (*P* = 0.020). Logistic regression adjusted for age and sex did not confirm the expected association of the *TMPRSS2* gene polymorphism (OR = 1.19, 95% CI 0.84-1.71, *P* = 0.331) with the severity of COVID-19. In patients with mild disease severity (Table 7), carriers of the risk GG genotype had significantly higher total bilirubin levels (*P* = 0.042) than carriers of the AA genotype. Carriers of the AA genotype had significantly higher urea levels (*P* = 0.020) and lower hematocrit levels (*P* = 0.028) than carriers of the AG genotype. In patients with moderate disease severity (Table 8), carriers of the high-risk GG genotype exhibited significantly higher CK levels (*P* = 0.022) than those with the AA genotype. Patients with the AA genotype displayed significantly lower CK levels (*P* = 0.010) than those with the AG genotype. Among the COVID-19 patients with severe clinical outcomes (Table 9), carriers of the high-risk GG genotype demonstrated significantly higher potassium levels (*P* = 0.003) than those with the AA genotype. Patients with the AA genotype showed notably lower potassium levels (*P* = 0.040) than those with the AG genotype.

**Table 7:** Comparison of clinical and biochemical characteristics between different genotypes of *TMPRSS2* gene polymorphism rs2070788 in COVID-19 patients with mild disease severity*^†^

	TMPRSS2 rs2070788 A>G					
	AA (n = 83)	AG (n = 116)	GG (n = 63)	*P* value^‡^ AA/AG	*P* value^‡^ AA/GG	*P* value^‡^ AG/GG
Leukocytes (10^9^/L)	6.87 ± 2.89	6.89 ± 3.65	6.28 ± 2.67	1.000	0.673	0.853
Erythrocytes (10^12^/L)	4.35 ± 0.51	4.49 ± 0.50	4.40 ± 0.55	0.167	1.000	0.705
Hemoglobin (g/L)	131.06 ± 19.47	137.23 ± 20.40	136.49 ± 17.93	1.000	0.894	1.000
Hematocrit (%)	37.58 ± 5.16	39.41 ± 4.63	38.87 ± 5.11	**0.028**	0.356	1.000
ESR (mm/h)	58.86 ± 41.30	62.40 ± 39.35	63.18 ± 39.71	1.000	1.000	1.000
Platelet count (10^9^/L)	225.49 ± 92.53	201.97 ± 70.26	215.95 ± 87.23	0.214	1.000	0.898
aPTT (sec)	28.22 ± 5.91	28.51 ± 4.86	28.05 ± 8.92	1.000	1.000	0.874
PT (sec)	13.39 ± 3.35	13.29 ± 3.03	13.20 ± 2.17	1.000	1.000	1.000
INR	1.12 ± 0.35	1.12 ± 0.26	1.11 ± 0.27	1.000	1.000	1.000
CRP (mg/L)	52.40 ± 69.56	68.42 ± 78.03	45.73 ± 51.90	0.084	1.000	0.191
D dimer (mg/L)	1.12 ± 1.33	1.36 ± 1.63	1.58 ± 1.68	0.931	0.358	1.000
Glucose (mmol/L)	7.34 ± 3.57	8.34 ± 4.52	8.09 ± 4.17	0.182	0.454	1.000
Urea (mmol/L)	7.58 ± 3.68	6.44 ± 2.48	6.59 ± 2.53	**0.020**	0.130	1.000
Creatinine (umol/L)	92.93 ± 57.39	89.43 ± 22.44	93.97 ± 27.65	1.000	0.708	0.806
Total bilirubin (umol/L)	14.02 ± 8.14	13.65 ± 4.51	15.96 ± 8.22	1.000	**0.042**	0.063
CK (IU/L)	155.96 ± 162.06	338.34 ± 856.37	339.59 ± 855.62	0.415	0.151	1.000
LDH (IU/L)	449.06 ± 188.49	504.72 ± 234.67	465.98 ± 204.52	0.119	1.000	0.776
Sodium (mmol/L)	139.88 ± 14.74	141.63 ± 4.23	141.86 ± 3.75	0.486	0.607	1.000
Potassium (mmol/L)	4.33 ± 0.57	4.24 ± 0.49	4.38 ± 0.55	0.942	1.000	0.326
sO_2_ (%)	89.26 ± 6.94	90.17 ± 6.45	90.21 ± 6.15	1.000	1.000	1.000
ctO_2c_ (vol%)	18.17 ± 3.03	19.15 ± 2.30	18.95 ± 2.63	**0.044**	0.276	1.000
FMetHb (%)	1.27 ± 0.19	1.25 ± 0.22	1.28 ± 0.19	1.000	1.000	0.842

*Abbreviations: ESR – erythrocyte sedimentation rate; aPTT – activated partial thromboplastin time; PT – prothrombin time; INR – international normalized ratio; CRP – C-reactive protein; CK – creatine kinase; LDH – lactate dehydrogenase; sO_2_ – oxygen saturation; ctO2c – sum of oxygen solved in plasma and chemically bound to hemoglobin; FMetHb – fraction of total hemoglobin that is present as methemoglobin (MetHb).

†Values represent mean ± standard deviation.

‡All differences between various genotypes were tested using ANOVA test (Bonferroni post hoc).

**Table 8:** Comparison of clinical and biochemical characteristics between different genotypes of the *TMPRSS2* gene polymorphism rs2070788 in COVID-19 patients with moderate disease severity*^†^

	TMPRSS2 rs2070788 A>G					
	AA (n = 53)	AG (n = 135)	GG (n = 64)	*P* value AA/AG^‡^	*P* value AA/GG^‡^	*P* value AG/GG^‡^
Leukocytes (10^9^/L)	8.21 ± 3.82	6.90 ± 3.33	7.32 ± 4.01	0.073	0.416	1.000
Erythrocytes (10^12^/L)	4.33 ± 0.51	4.34 ± 0.54	4.23 ± 0.46	1.000	0.864	0.431
Hemoglobin (g/L)	132.88 ± 20.55	131.73 ± 17.82	130.94 ± 14.16	1.000	1.000	1.000
Hematocrit (%)	37.92 ± 5.51	37.92 ± 5.04	37.53 ± 4.06	1.000	1.000	1.000
ESR (mm/h)	77.82 ± 48.06	74.91 ± 37.98	71.12 ± 37.18	1.000	1.000	1.000
Platelet count (10^9^/L)	217.27 ± 84.44	220.26 ± 96.71	208.88 ± 82.22	1.000	1.000	1.000
aPTT (sec)	27.56 ± 10.03	28.00 ± 4.72	27.48 ± 4.55	0.580	1.000	1.000
PT (sec)	13.88 ± 3.59	13.75 ± 3.08	13.51 ± 2.12	1.000	1.000	1.000
INR	1.18 ± 0.34	1.16 ± 0.34	1.13 ± 0.22	1.000	1.000	1.000
CRP (mg/L)	120.10 ± 153.86	92.70 ± 77.35	83.86 ± 76.69	1.000	0.151	0.207
D dimer (mg/L)	2.91 ± 3.32	2.06 ± 2.32	2.22 ± 2.55	1.000	1.000	1.000
Glucose (mmol/L)	8.92 ± 4.98	8.70 ± 4.47	7.75 ± 3.66	1.000	0.330	0.439
Urea (mmol/L)	8.94 ± 6.03	8.51 ± 4.99	8.18 ± 3.88	1.000	1.000	1.000
Creatinine (umol/L)	98.56 ± 42.17	97.54 ± 39.57	97.60 ± 38.99	1.000	1.000	1.000
Total bilirubin (umol/L)	17.74 ± 17.57	14.31 ± 6.78	16.76 ± 12.45	0.559	1.000	0.290
CK (IU/L)	135.23 ± 163.49	242.25 ± 361.41	257.17 ± 468.88	**0.010**	**0.022**	0.963
LDH (IU/L)	582.96 ± 306.93	562.73 ± 212.50	606.84 ± 324.90	1.000	1.000	1.000
Sodium (mmol/L)	141.04 ± 4.75	140.45 ± 12.34	140.66 ± 3.73	1.000	1.000	1.000
Potassium (mmol/L)	4.31 ± 0.80	4.31 ± 0.63	4.35 ± 0.56	1.000	1.000	1.000
sO_2_ (%)	88.99 ± 5.62	87.49 ± 7.97	89.58 ± 5.97	0.573	1.000	0.204
ctO_2c_ (vol%)	18.29 ± 2.98	18.11 ± 3.47	18.07 ± 2.41	0.059	0.796	0.776
FMetHb (%)	1.32 ± 0.27	1.23 ± 0.23	1.27 ± 0.26	1.000	1.000	1.000

*Abbreviations: ESR – erythrocyte sedimentation rate; aPTT – activated partial thromboplastin time; PT – prothrombin time; INR – international normalized ratio; CRP – C-reactive protein; CK – creatine kinase; LDH – lactate dehydrogenase; sO_2_ – oxygen saturation; ctO2c – sum of oxygen solved in plasma and chemically bound to hemoglobin; FMetHb – fraction of total hemoglobin that is present as methemoglobin (MetHb).

†Values represent mean ± standard deviation.

‡All differences between various genotypes were tested using ANOVA test (Bonferroni post hoc).

**Table 9:** Comparison of clinical and biochemical characteristics between different genotypes of TMPRSS2 gene polymorphism rs2070788 in COVID-19 patients with severe clinical outcome*^†^

	TMPRSS2 rs2070788 A>G					
	AA (n = 68)	AG (n = 102)	GG (n = 65)	*P* value^‡^ AA/AG	*P* value^‡^ AA/GG	*P* value^‡^ AG/GG
Leukocytes (10^9^/L)	7.67 ± 3.83	7.73 ± 4.28	7.54 ± 4.27	1.000	1.000	1.000
Erythrocytes (10^12^/L)	4.27 ± 0.62	4.33 ± 0.59	4.26 ± 0.67	1.000	1.000	1.000
Hemoglobin (g/L)	133.22 ± 20.45	132.47 ± 19.11	132.92 ± 20.26	1.000	1.000	1.000
Hematocrit (%)	37.97 ± 5.86	37.94 ± 5.39	38.09 ± 5.98	1.000	1.000	1.000
ESR (mm/h)	75.22 ± 40.56	74.40 ± 42.26	75.86 ± 39.27	1.000	1.000	1.000
Platelet count (10^9^/L)	186.88 ± 85.31	195.34 ± 79.78	197.05 ± 99.54	1.000	1.000	1.000
aPTT (sec)	28.25 ± 5.14	28.29 ± 4.31	28.61 ± 5.12	1.000	1.000	1.000
PT (sec)	17.00 ± 18.03	14.96 ± 15.39	15.99 ± 18.48	0.314	1.000	1.000
INR	1.21 ± 0.35	1.11 ± 0.26	1.13 ± 0.29	0.068	0.308	1.000
CRP (mg/L)	116.50 ± 84.83	122.13 ± 89.45	124.88 ± 105.33	1.000	1.000	1.000
D dimer (mg/L)	2.49 ± 2.70	2.00 ± 2.07	2.08 ± 2.22	0.824	1.000	1.000
Glucose (mmol/L)	9.29 ± 4.89	9.41 ± 4.77	10.31 ± 7.96	1.000	1.000	1.000
Urea (mmol/L)	10.39 ± 6.68	10.21 ± 6.45	11.48 ± 8.64	1.000	1.000	1.000
Creatinine (umol/L)	111.72 ± 61.10	128.25 ± 82.88	141.15 ± 136.28	0.384	0.437	1.000
Total bilirubin (umol/L)	20.40 ± 37.17	17.69 ± 19.28	14.80 ± 4.64	1.000	0.881	1.000
CK (IU/L)	422.24 ± 911.87	320.83 ± 453.29	340.03 ± 593.89	1.000	1.000	1.000
LDH (IU/L)	690.65 ± 392.93	728.71 ± 525.19	824.37 ± 834.31	1.000	0.447	0.758
Sodium (mmol/L)	137.37 ± 16.73	141.42 ± 10.83	139.35 ± 4.26	0.162	0.594	1.000
Potassium (mmol/L)	4.15 ± 0.64	4.38 ± 0.66	4.61 ± 0.87	**0.040**	**0.003**	0.226
sO_2_ (%)	83.88 ± 10.54	83.86 ± 9.13	83.53 ± 10.93	1.000	1.000	1.000
ctO_2c_ (vol%)	17.13 ± 3.64	16.71 ± 3.33	17.17 ± 3.44	1.000	1.000	1.000
FMetHb (%)	1.29 ± 0.24	1.26 ± 0.19	1.24 ± 0.28	1.000	0.322	0.948

*Abbreviations: ESR – erythrocyte sedimentation rate; aPTT – activated partial thromboplastin time; PT – prothrombin time; INR – international normalized ratio; CRP – C-reactive protein; CK – creatine kinase; LDH – lactate dehydrogenase; sO_2_ – oxygen saturation; ctO2c – sum of oxygen solved in plasma and chemically bound to hemoglobin; FMetHb – fraction of total hemoglobin that is present as methemoglobin (MetHb).

†Values represent mean ± standard deviation.

‡All differences between various genotypes were tested using ANOVA test (Bonferroni post hoc).

## Discussion

Our research revealed significant associations between genetic variants in the *ACE2* and *TMPRSS2* genes and various clinical, biochemical, and hematological parameters in individuals affected by COVID-19, who were categorized into different clinical outcomes. Furthermore, the genotype frequencies of the *TMPRSS2* gene polymorphism showed significant disparities among the mild, moderate, and severe patient groups, which suggests a potential correlation between this genetic variant and COVID-19 severity. These results can help us understand the intricate relationship between COVID-19 severity and various physiological markers. The discernible variations in these parameters provide valuable insights into the physiological impacts of the disease and could potentially aid in refining risk stratification and treatment strategies for patients with differing clinical outcomes. It is important to note that the ACE2 gene polymorphism (rs2285666) deviated from Hardy-Weinberg equilibrium, while the TMPRSS2 gene polymorphism (rs2070788) was in HWE. Other studies also reported that the ACE2 gene polymorphism deviated from HWE (16). This observation could potentially be attributed to the pivotal role and significant function of this gene in facilitating virus entry into cells. However, confirming this hypothesis necessitates a healthy control group unaffected by COVID-19.

In recent studies, *ACE2* and *TMPRSS2* have emerged as potential candidate genes associated with the development of COVID-19 (5,17). The present study is one of only two that have specifically targeted these two genes to assess their impact on SARS-CoV-2 infection and their correlation with the severity of the clinical presentation within the population of Bosnia and Herzegovina (18). Also, only a limited number of studies have delved into the influence of these genetic variations on important biochemical parameters. Additionally, we explored the genetic aspect of COVID-19 susceptibility by examining the allele and genotype frequencies of specific gene polymorphisms. Examination of the genotype frequencies of the *ACE2* gene polymorphism did not reveal any significant differences among the three groups. On the other hand, the genotype frequencies of the *TMPRSS2* gene polymorphism showed significant differences between the mild and moderate patient groups. Logistic regression did not find the expected associations between the *ACE2* and *TMPRSS2* gene polymorphisms and the COVID-19 severity, perhaps due to the limited number of participants in our study. Several studies conducted across diverse populations have consistently demonstrated the influence of these genetic variants in elevating the risk of severe cases of COVID-19 (19-23). However, certain studies conducted within Spanish, German, and Turkish populations did not confirm this association (24-26).

The analysis of ACE2 gene polymorphism rs2285666 showed significant findings. Individuals with the risk TT genotype displayed elevated aPTT and INR, which suggests a link between this genetic variant and coagulation changes. Interestingly, TT genotype carriers exhibited lower LDH levels, which implies distinct disease mechanisms. The study by Abdelsattar et al, who combined CT and TT genotypes in one group, revealed a similarity in their impact on coagulation factors, particularly D-dimer and platelet levels, as well as the influence of the risk T allele on LDH levels (19). Furthermore, our results showed that the risk TT genotype individuals had higher creatinine levels than CC genotype carriers, who showed elevated hemoglobin, hematocrit, creatinine, and CK levels. This contradicts the findings of Abdelsattar et al (19), who found that individuals with the CC genotype exhibited lower hemoglobin and hematocrit values than those with the CT+TT genotypes. A potential explanation for the contradictory results could stem from differences in methodology. The study by Abdelsattar et al (19) had a healthy control group, whereas our study had twice as many participants. Furthermore, Möhlendick et al suggested that the CC genotype was linked to more severe COVID-19 outcomes, while the TT genotype might play a potentially “protective” role (22). In our investigation of the *TMPRSS2* gene polymorphism rs2070788 among patients with mild disease severity, we also showed significant links to hematological and biochemical markers. Carriers of the risk GG genotype exhibited significantly higher total bilirubin levels than AA genotype carriers. Conversely, individuals with the AA genotype displayed significantly elevated urea levels and lower hematocrit levels than AG genotype carriers. These findings highlight the intricate interplay between genetic variations and physiological responses in mild COVID-19 cases. Liver involvement in COVID-19 varies widely, ranging from asymptomatic increases in liver function tests to severe hepatic decompensation. Abnormal liver test results have been linked to more severe COVID-19 and higher mortality rates. Additionally, severe cases of COVID-19 have shown the presence of viral RNA in the liver. Expression data from the human liver cell atlas indicates TMPRSS2 protease expression, primarily in cholangiocytes and, to a lesser extent, in hepatocytes, suggesting SARS-CoV-2 entry into parenchymal cells. Experimental evidence further supports SARS-CoV-2 replication in liver organoids (27). Clinical data reports liver injury in 14.8%-53% of COVID-19 patients during viremia or the inflammatory phase, which can potentially increase bilirubin levels when combined with TMPRSS2 expression (28).

Also, we explored potential genetic associations between specific gene polymorphisms and crucial blood parameters, with a particular focus on patients with moderate and severe disease severity. These findings offer valuable insights into the genetic underpinnings of physiological responses during COVID-19 infection. In patients with moderate disease severity, a significant relationship emerged between the *ACE2* gene polymorphism rs2285666 and various blood parameters. Notably, individuals carrying the high-risk TT genotype of rs2285666 displayed distinctive blood profiles compared with those with the CC genotype. TT genotype carriers exhibited significantly increased FMetHb levels, which suggests a potential genetic influence on oxidative stress or oxygen-carrying capacity. They also showed elevated erythrocyte counts, hemoglobin levels, hematocrit levels, and aPTT levels, which implies that genetic variations may impact red blood cell production, blood viscosity, and coagulation mechanisms. In contrast, CC genotype individuals had significantly higher erythrocyte counts and hemoglobin levels while displaying lower ESR values and FMetHb levels compared with CT genotype carriers, which highlights diverse physiological responses associated with different ACE2 genotypes. SARS-CoV-2 infection significantly disrupts the structural membrane balance of red blood cells (RBCs) at the protein and lipid levels. RBCs in COVID-19 patients display elevated glycolytic intermediates and experience oxidative damage and protein fragmentation. Consequently, COVID-19 affects two crucial mechanisms governing RBC membranes and hemoglobin's oxygen affinity. These altered RBCs may struggle to adapt to varying oxygen levels during circulation, which impairs oxygen transport. Hematological abnormalities are common in COVID-19, with reductions in platelets, lymphocytes, hemoglobin, eosinophils, and basophils observed early in the disease, correlating with disease severity and outcomes (29). Our investigation of COVID-19 patients with moderate disease severity also revealed a significant association between the *TMPRSS2* gene polymorphism rs2070788 and CK levels. High-risk GG genotype carriers had significantly elevated CK levels compared with AA genotype individuals, whereas AA genotype individuals exhibited notably lower CK levels than AG genotype carriers. These findings highlight the role of genetic variations in influencing CK levels and their potential implications for the pathophysiology of COVID-19 in moderately affected patients.

In patients with severe clinical outcomes, we observed significant genetic associations involving the *ACE2* gene polymorphism rs2285666. TT genotype carriers displayed significantly higher erythrocyte counts, hemoglobin levels, and hematocrit levels than CC and CT genotype carriers. Conversely, CC genotype individuals exhibited higher hemoglobin and hematocrit levels and lower glucose levels than CT genotype carriers. These findings underscore genetic heterogeneity in severe COVID-19 cases, suggesting that genetic variations may contribute to diverse blood parameter profiles and potentially influence disease outcomes. In an Iranian study, the CC genotype was associated with a higher mortality rate. Additionally, creatinine, ESR, and CRP were also associated with an increased risk of mortality in COVID-19 patients (30). In our study, in severe patient subgroup, the *TMPRSS2* gene polymorphism rs2070788 was significantly associated with specific blood parameters. High-risk GG genotype carriers had significantly higher potassium levels than AA genotype individuals, while AA genotype individuals displayed lower potassium levels than AG genotype carriers. Pandey et al (23) pointed to the association of the G allele with complications and a higher case fatality rate in the Indian population, similar to the Brazilian population, where the GG genotype was associated with severe forms of COVID-19 (31). In agreement with this finding, a recent Dutch study of 188 adult hospitalized patients demonstrated a protective effect of the rs2070788 AA genotype on COVID-19 severity (32). Also, in older individuals, who have elevated TMPRSS2 levels, the rs2070788 GG genotype in *TMPRSS2* worsened ACE2 cleavage, potentially determining clinical outcomes (31). We also found a significant association of *TMPRSS2* polymorphism with potassium levels in severe cases of SARS-CoV-2 infection. SARS-CoV-2 disrupts potassium balance by activating epithelial sodium channels (ENaC) due to TMPRSS2's role in viral entry. ENaC hyperactivity depletes intracellular potassium, driving TMPRSS2 expression. Normally, TMPRSS2 inhibits ENaC, but in COVID-19, ENaC remains active as TMPRSS2 aids viral entry (33).

In conclusion, our findings shed light on the intricate interplay between genetic factors, biochemical markers, and disease severity in COVID-19. While certain genetic polymorphisms exhibited deviations from HWE and genotype frequency variations were observed, the adjusted odds ratios suggest a more complex relationship between these genetic factors and the clinical outcomes of COVID-19 than initially anticipated. However, it is important to acknowledge the limitations of this study, including the relatively small sample size. Validation in larger and more diverse populations is necessary. Additionally, the study lacked a healthy control group, and stratification based on underlying diseases and sex, which are crucial factors that should be considered. Collecting a larger number of patients was challenging due to the relatively small overall population in Bosnia and Herzegovina, as well as limitations within our health care system and its organization. Additionally, the samples were collected during the COVID-19 pandemic, when the focus was on the health and survival of patients, leading to overcrowded hospitals. Therefore, it was not possible to gather a larger number of patients and obtain a complete picture, especially concerning comorbidities, which considerably affect the mortality of COVID-19 patients. Further research and functional studies are warranted to decipher the underlying biological mechanisms behind these observed correlations. The correlations observed in *ACE2* and *TMPRSS2* gene polymorphisms with diverse hematological and biochemical markers offer potential avenues for further exploration into disease progression mechanisms. While the precise mechanisms remain elusive, our study contributes to the growing body of evidence emphasizing genetic factors in disease outcomes.
